# Surface characterization of nanoparticles using near-field light scattering

**DOI:** 10.3762/bjnano.9.114

**Published:** 2018-04-18

**Authors:** Eunsoo Yoo, Yizhong Liu, Chukwuazam A Nwasike, Sebastian R Freeman, Brian C DiPaolo, Bernardo Cordovez, Amber L Doiron

**Affiliations:** 1 Department of Biomedical Engineering, Binghamton University (SUNY), P.O. Box 6000 Binghamton, NY 13902, USA,; 2 Optofluidics, Inc., 3711 Market St. Philadelphia, PA 19104, USA

**Keywords:** nanoparticle surface properties, nanoparticles, nanophotonic force microscopy, near-field light scattering, superparamagnetic iron oxide

## Abstract

The effect of nanoparticle surface coating characteristics on colloidal stability in solution is a critical parameter in understanding the potential applications of nanoparticles, especially in biomedicine. Here we explored the modification of the surface of poly(ethylene glycol)-coated superparamagnetic iron oxide nanoparticles (PEG-SPIOs) with the synthetic pseudotannin polygallol via interpolymer complexation (IPC). Changes in particle size and zeta potential were indirectly assessed via differences between PEG-SPIOs and IPC-SPIOs in particle velocity and scattering intensity using near-field light scattering. The local scattering intensity is correlated with the distance between the particle and waveguide, which is affected by the size of the particle (coating thickness) as well as the interactions between the particle and waveguide (related to the zeta potential of the coating). Therefore, we report here the use of near-field light scattering using nanophotonic force microscopy (using a NanoTweezer^TM^ instrument, Halo Labs) to determine the changes that occurred in hydrated particle characteristics, which is accompanied by an analytical model. Furthermore, we found that altering the salt concentration of the suspension solution affected the velocity of particles due to the change of dielectric constant and viscosity of the solution. These findings suggest that this technique is suitable for studying particle surface changes and perhaps can be used to dynamically study reaction kinetics at the particle surface.

## Introduction

Nanotechnology is an increasingly integral part of modern medicine, predominantly in the fields of cell labeling, gene and drug delivery, molecular imaging, and biosensors [1–4]. Nanoparticles can be manipulated or modified to fulfill a specific, engineered purpose, partially through changing the surface chemistry or surface coating of the particle. As such, changes in particle surface properties are often important in determining whether the particles are appropriate for use in various applications [5–16]. Due to the difficulty in predicting nanoparticle behavior in a biological environment, there is a need for newer techniques to evaluate changes at the particle surface in varied solvents [7,17–18]. In this work, we present one potential application of near-field light scattering-based nanophotonic force microscopy used to evaluate changes in particle surface and size by examining the self-assembly of interpolymer complexed superparamagnetic iron oxide nanoparticles (IPC-SPIOs).

**Figure 1:**
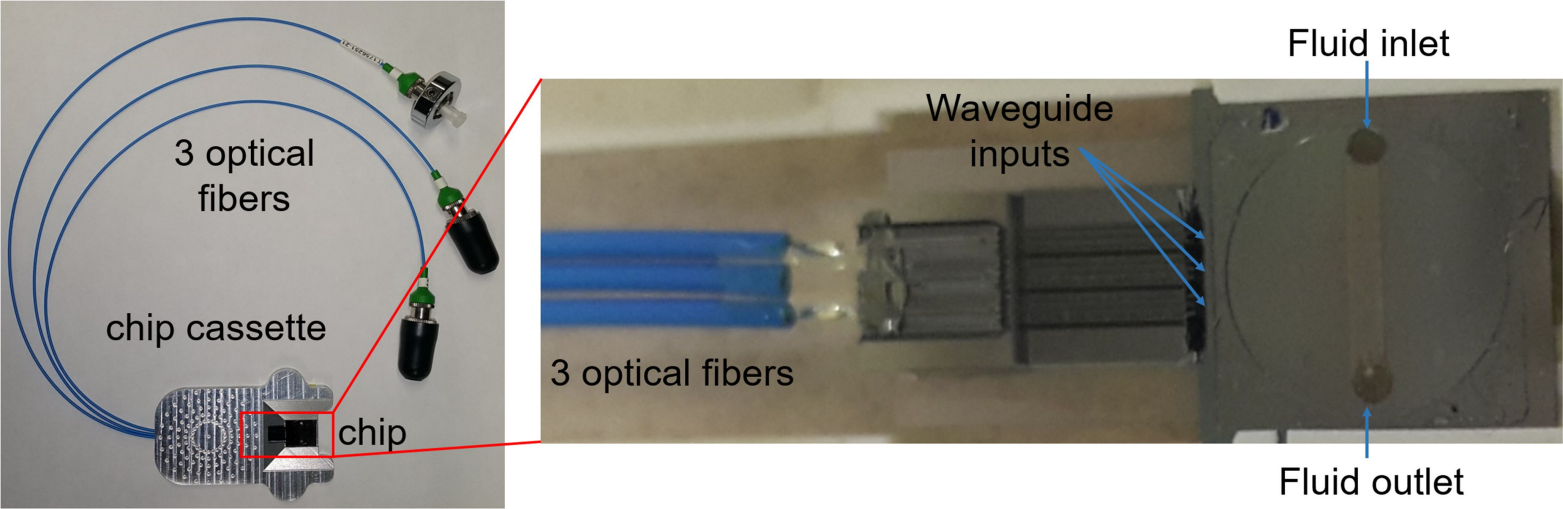
Optofluidic chip is secured in a chip cassette and is permanently bonded to three optical fibers, which deliver laser light to the three waveguides on the chip surface. The chip also has a microfluidic channel with inlet and outlet holes.

Nanophotonic force microscopy pushes particles against a waveguide surface, optically trapping the particles by light confinement [10,19–20]. The evanescent fields are created by the waveguide, and there are four forces operating on the field: the gradient force (trapping force), scattering force, coating force, and drag force (Figure 1) [21]. Nanoparticles are either trapped in the evanescent field and reside in a potential well or escape the potential well via Brownian motion due to inadequate trapping force [22]. The potential well is the sum of all forces, and it is generated by both the optical trapping force and surface repulsion of the nanoparticle [23]. The motion and behavior, i.e., velocity and local scattering intensity, of trapped nanoparticles is characteristic of the particle properties. When nanoparticles are trapped, the local refractive index of the waveguide is changed, which produces a local increase in intensity, and high-speed imaging of trapped nanoparticles allows for measurement of interactions between trapped nanoparticles and the waveguide. A higher intensity signal is created by a particle in close proximity to the waveguide, while a lower intensity signal results when the particle is located a greater distance away from the waveguide. The local intensity is used to determine the particle equilibrium height (repulsion distance), potential energy (scattering force), and contact surface force (gradient force) for individually trapped nanoparticles. Additionally, unlike most techniques available today, nanophotonic force microscopy measures the velocity of nanoparticles to determine interactions between the waveguide and particles that are correlated with the surface properties of individual particles.

A recent study by Hristov and colleagues established that near-field light scattering using the nanophotonic force microscope can be used to measure the size of bare silica and PEG-coated silica nanoparticles very accurately [24]. PEGylation of the nanoparticle slightly altered the particle’s surface chemistry and increased the attraction between the nanoparticle and waveguide. Researchers attributed the increased diffusion of PEGylated particles towards the waveguide to the slight reduction in particle surface charge (zeta potential) after PEGylation [24]. This study provided evidence that the nanophotonic force microscope is efficient in characterization of polymer-coated nanoparticles. However, due to the fact that the nanoparticle scattering intensity depends on the dielectric constant of the material, multicomponent particle coatings, especially those on metallic or metal oxide materials such as those studied here, make finite measurement of the particle size more arduous with nanophotonic force microscopy. Here we report a simpler method to determine changes in particle characteristics based on particle velocity analysis.

Particles that have been coated with complexed polymers travel at a different velocity when compared to uncoated/single layer coated nanoparticles, complicating the analysis of the size of such particles. The surface properties and size collectively determine the interaction of the particles with the waveguide and detected local intensity. To this end, three classes of particles, namely uncoated superparamagnetic iron oxide nanoparticles, polyethylene glycol-coated superparamagnetic iron oxide nanoparticles, and interpolymer complex-superparamagnetic iron oxide nanoparticles, were studied for changes in collective size and surface properties using an analysis of particle velocity on the waveguide.

Superparamagnetic iron oxide nanoparticles (SPIOs) have been extensively studied due to their unique chemical, physical, thermal, and mechanical applications in areas such as cell labeling, tissue repair, drug delivery, magnetic resonance (MR) contrast, and hyperthermia [25–29]. The properties of SPIOs vary considerably based on the particle size and surface coating, properties, which also greatly affect the particles’ in vivo biodistribution and effectiveness in biological applications [30–31]. During circulation, it is generally understood that larger nanoparticles have a shorter biological half-life and are cleared by the reticuloendothelial system [32]. For biomedical purposes, SPIOs must be biocompatible, colloidally stable, and well-dispersed in physiological buffers, which is often accomplished by surface modification of the magnetic nanoparticles with biocompatible polymers such as poly(ethylene glycol) (PEG) [32–33]. PEG and PEG-containing copolymers tend to increase the circulation time of nanoparticles by reducing opsonization [32,34]. A polymeric pseudotannin has been designed to mimic natural tannin structures and form nanoscale interpolymer complexes with PEG via hydrogen bonding as a novel coating for SPIO magnetic resonance contrast agents [35–37]. The stability of these complexes is highly dependent on the plurality of the hydrogen bonds between PEG and pseudotannin [35–36]. In this study, photonic chips were provided by Optofludics, Inc. (Figure 1).

**Figure 2:**
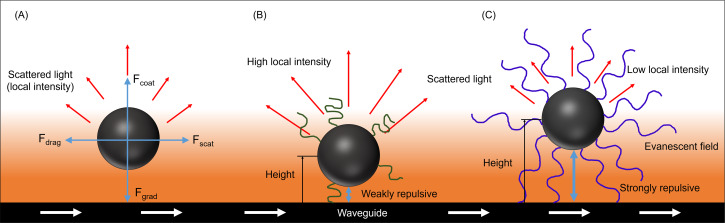
(A) Near-field light scattering with relevant forces acting on the particle. (B, C) Trapped particles with different surface properties move along the waveguide with an equilibrium height determined by the balance of surface repulsion and optical trapping forces.

We hypothesized that the complexation of PEG-SPIOs and pseudotannin would greatly change the surface properties of the particles, and we used nanophotonic force microscopy to observe this change (Figure 2). Furthermore, recent work by Kong et al. demonstrated the combination of nanophotonic force microscopy combined with surface enhanced Raman spectroscopy (SERS) to enable trapping and chemical characterization of individual metallic nanoparticles [38]. Although not done in the present study, a combined approach using our methods and those of Kong et al. may be a powerful tool for dynamic studies of reaction kinetics at the particle surface that result in changes in particle size, dielectric constant, or surface chemistry, which is of particular interest in protein adsorption to nanoparticles for biomedical applications.

## Results and Discussion

### Size, morphology, and elemental analysis of nanoparticles

**Figure 3:**
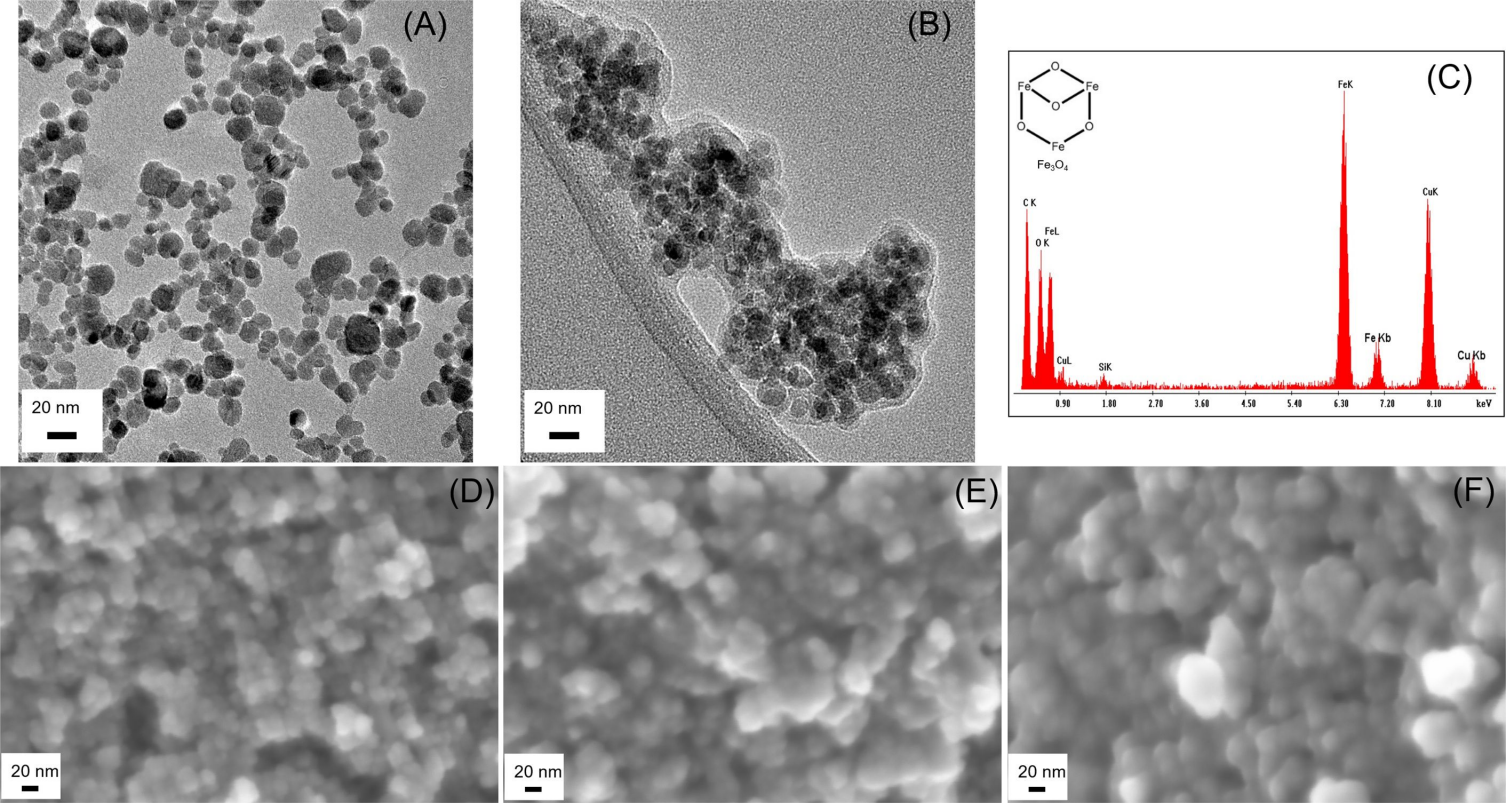
TEM images of uncoated SPIOs (A), PEG-SPIOs (B) with scale bar of 20 nm. EDS spectrum of uncoated SPIOs (Fe_3_O_4_) (C). SEM images of uncoated SPIOs (D), PEG-SPIOs (E), and IPC-SPIOs with PEG and polygallol (F). PEG-SPIOs (*n* = 66) and uncoated SPIOs (*n* = 25) were measured to calculate the mean size of the particles and PEG coating thickness in the TEM images, respectively.

Transmission electron microscopy (TEM) was used to image uncoated SPIOs and PEG-SPIOs; the results are shown in Figure 3. Although the particles were aggregated as a result of the drying process, uncoated SPIOs exhibited spherical morphology (Figure 3A). After PEGylation, PEG-SPIOs showed a gray halo around the SPIO core as a result of PEGylation (Figure 3B). Using the TEM images, the mean and standard deviation of the particle size for uncoated SPIOs and the PEG coating thickness of PEG-SPIOs were measured to be 13 ± 3 nm (*n* = 66) and 4 ± 1.0 nm (*n* = 25), respectively. Images of the IPC-SPIOs were not obtainable because of the continuous degradation of the polymer coating under the electron beam exposure. The energy dispersive X-ray spectroscopy (EDS) spectrum of the uncoated SPIOs (Figure 3C) showed peaks corresponding to iron (Fe) and oxygen (O), as expected, while the TEM grid and Si (Li) detector caused peaks for carbon (C), copper (Cu), and silicon (Si). These EDS data did not show any additional elemental peaks from impurities.

Additionally, scanning electron microscopy (SEM) was used to image uncoated SPIOs, PEG-SPIOs, and IPC-SPIOs, as shown in Figure 3D–F. The particles were moderately spherical, with an average diameter of 18 ± 2 nm (*n* = 20) for uncoated SPIOs, 30 ± 6 nm (*n* = 20) for PEG-SPIOs, and 39 ± 5 nm (*n* = 20) for IPC-SPIOs. There was a gradual increase in size with the addition of the polymer coatings on the SPIOs.

### Hydrodynamic diameter and zeta potential of nanoparticles

Dynamic light scattering (DLS) was used to measure the hydrodynamic diameter, polydispersity index, and zeta potential (ζ). The mean hydrodynamic diameter of the samples increased with the addition of PEG and polygallol to the complexation. The polydispersity index of uncoated SPIOs was 0.16, which was greater than that of PEG-SPIOs and IPC-SPIOs, likely due to aggregation and polydispersity of the sample synthesized using the co-precipitation method. The zeta potential of the uncoated SPIOs is strongly negative (−50 mV), while the zeta potential of PEG-SPIOs is slightly higher (−45 mV) due to the neutral PEG shielding the SPIO core. The thicker coating layer of PEG-pseudotannin on IPC-SPIOs shielded the charge even more (−26 mV).

### Particle characterization via nanophotonic force microscopy

**Figure 4:**
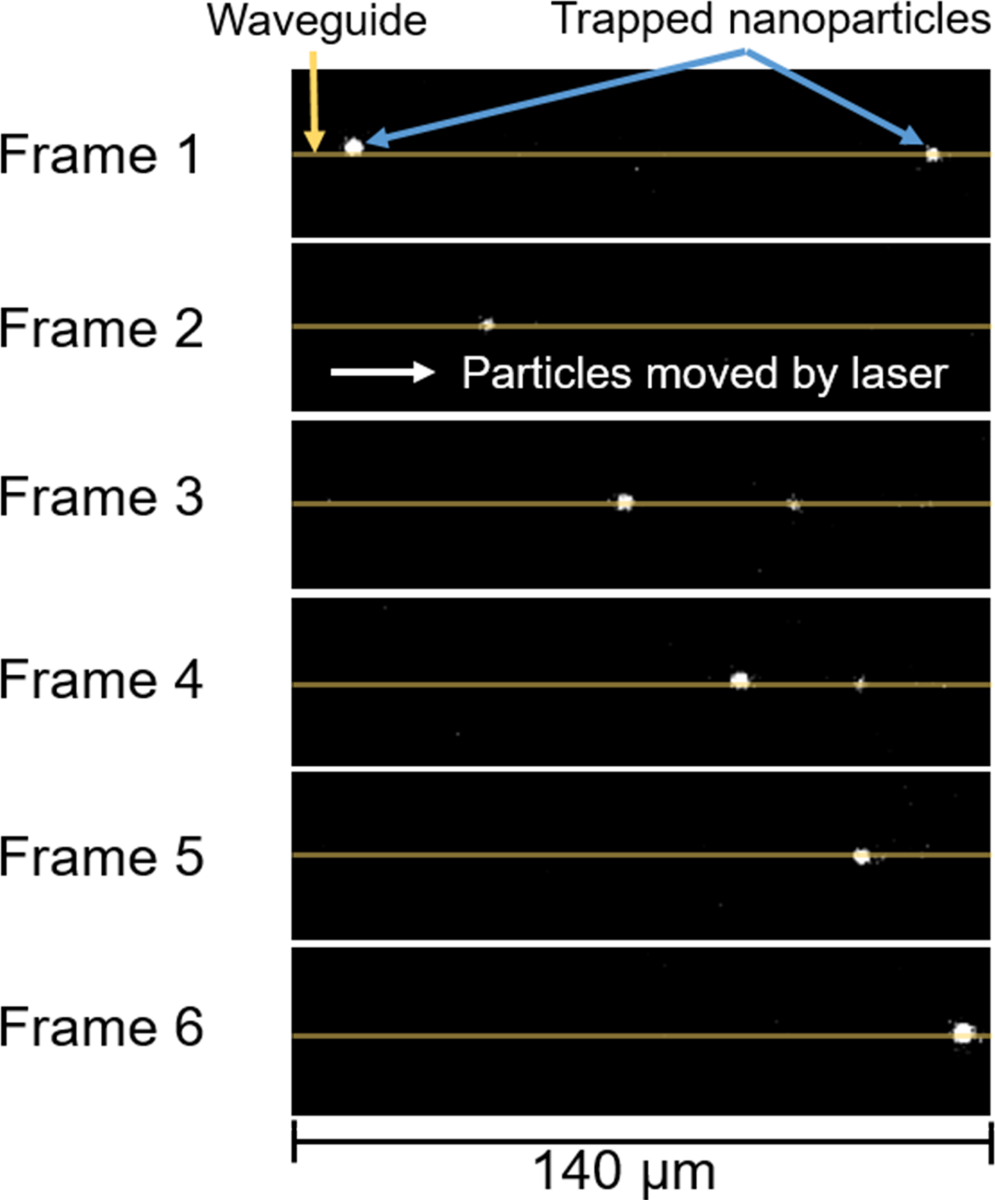
Microscopy images of PEG-SPIOs in aqueous solution. The laser pushes the trapped nanoparticles across the waveguide via scattering force (pushing force). The frames above capture the movement of trapped nanoparticles over different time points.

The NanoTweezer^TM^ system was used to analyze uncoated SPIOs, PEG-SPIOs, and IPC-SPIOs. Each particle sample was mixed with 1% pluronic (Pluronic F-68) before injection into the waveguide. The uncoated SPIOs stuck to the waveguide and could not be analyzed further, while the light scattered from PEG-SPIOs and IPC-SPIOs was imaged, as the laser pushed the trapped nanoparticles across the waveguide (Figure 4). The particle velocity across the waveguide was measured using successive frames, and the light intensity from scattering was also measured.

**Figure 5:**
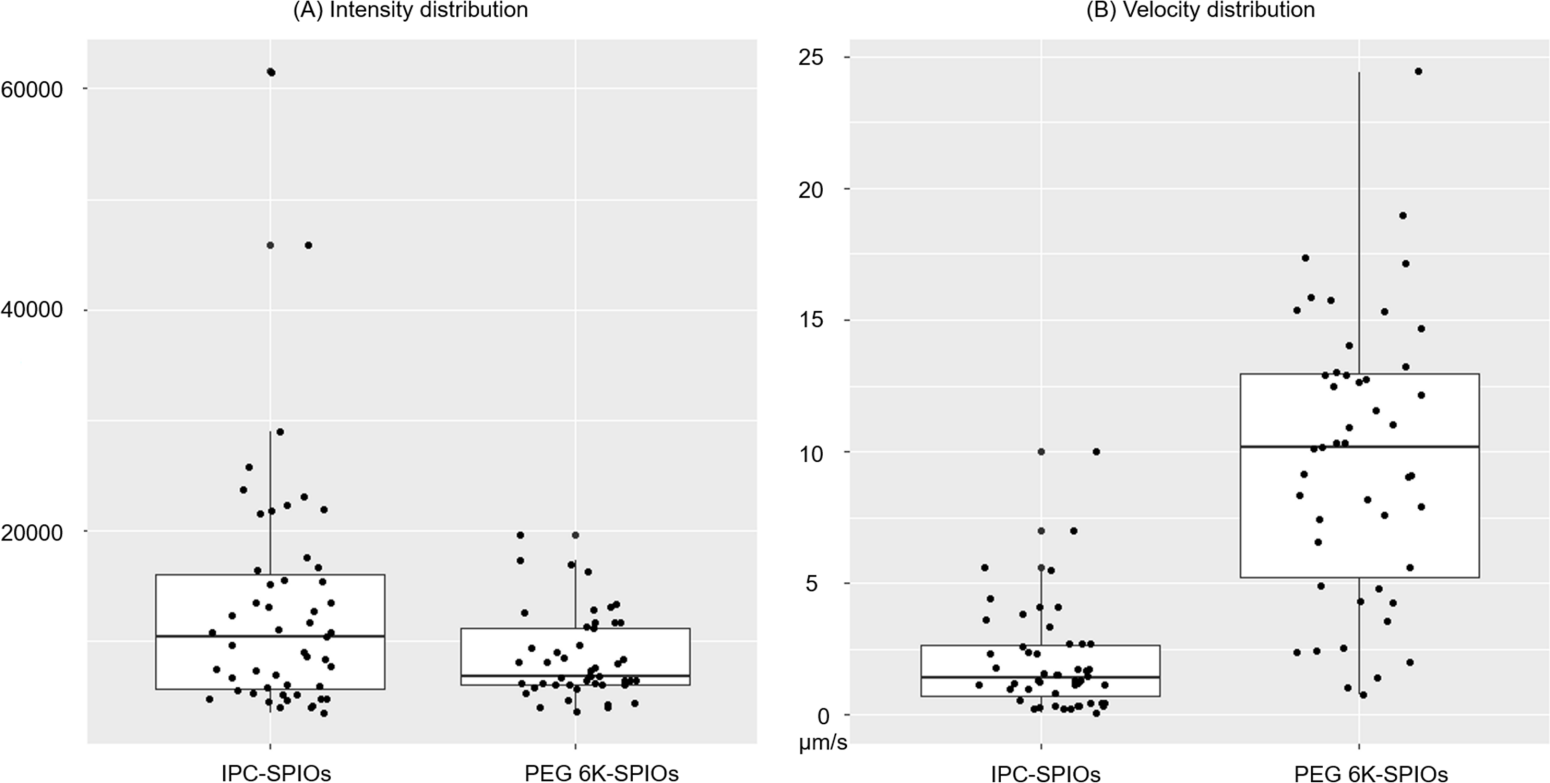
(A) Intensity and velocity distributions of PEG-SPIOs (*n* = 47) and IPC-SPIOs *n* = 51), presented as box plots.

For a given laser input power, the measured scattering intensity was similar for both PEG-SPIOs and IPC-SPIOs (Figure 5A), with a similar spread of the data. When the intensity measurements were used to calculate the size distributions using a Rayleigh scattering model that assumed that the lowest observed intensity corresponds to the particle diameter of a single uncoated SPIO (13 nm) measured by TEM, the calculated particle diameter for uncoated SPIOs, PEG-SPIOs, and IPC-SPIOs was very similar. However, this calculation neglects the difference in refractive index of the inorganic iron oxide nanoparticle core and the hydrated polymeric coating. Therefore, the particle diameter calculated for PEG-SPIOs and IPC-SPIOs with this method was not in agreement with the diameter measured using dynamic light scattering (Table 1).

**Table 1:** Hydrodynamic diameter, polydispersity index (PDI), and zeta potential (ζ) of uncoated SPIOs, PEG-SPIOs, IPC-SPIOs with PEG and polygallol measured in triplicate using a Malvern Zetasizer ZS instrument. The diameters also were measured by TEM and SEM.

	Diameter,DLS (nm)	PDI, DLS	ζ, DLS (mV)	Diameter, TEM (nm)	Diameter, SEM (nm)

Uncoated SPIOs	57.2 ± 2.7	0.161	−50.5 ± 0.5	12.95 ± 3.7 (*n* = 66)	18.3 ± 1.8 (*n* = 20)
PEG-SPIOs	72.6 ± 0.3	0.107	−45.6 ± 1.0	3.61 ± 1.0 (*n* = 25) (coating thickness)	30.2 ± 5.6 (*n* = 20)
IPC-SPIOs	112.9 ± 0.5	0.082	−26.0 ± 0.3	–	38.8 ± 5.2 (*n* = 20)

The distribution of velocity of individual particles (in micrometers per second (Figure 5B)) revealed that PEG-SPIOs moved significantly faster across the waveguide than IPC-SPIOs and also had a wider distribution in velocity. The spread of the velocity data amongst individual PEG-SPIO particles agrees with the larger polydispersity index measured for PEG-SPIOs as compared to IPC-SPIOs (Table 1). IPC-SPIOs likely experienced increased physical interaction with the waveguide surface compared with PEG-SPIOs, thereby decreasing their velocity, as is explored in the analytical model below. The increased interaction observed suggested that IPC-SPIOs were also less colloidally stable. According to the zeta potential data (Table 1), uncoated SPIOs and PEG-SPIOs have a highly negative charge, −50 mV and −45 mV, which is capable of initiating a strong electrostatic repulsion between the nanoparticles and the negatively charged waveguide. However, unlike PEG-SPIOs, uncoated SPIOs likely stuck to the waveguide owning to the absence of surface modification. Without a surface coating, which acts like a lubricant or spring, the uncoated SPIOs are attracted to the waveguide by the gradient force (attraction force) without the repulsive coating force to counterbalance the interaction. Moreover, IPC-SPIOs with a charge of −26 mV have a weaker electrostatic repulsion, which contributes to slower movement when compared to PEG-SPIOs. The details of these interactions are explored in the analytical model that follows.

### Analytical model for velocity vs intensity

We have developed a preliminary model to describe the effect of surface coating on a nanoparticle’s motion across the waveguide. As an example, we consider a particle and waveguide under an exponentially decaying repulsive force (e.g., electrostatic repulsion). We begin by equating the electrostatic repulsion force between the waveguide and coated nanoparticle, which exponentially decays as distance from the surface is increased, *F*_coat_* = F*_0_ exp(−γ*z*), with the optical trapping force, *F*_trap_ *=* [(2πα/*c*)·(∂*I*/∂*z*)], where *z* is the particle–waveguide separation, *F*_0_ is the force required to completely compress the coating whereby the particle touchs the waveguide, γ is the *F*_coat_ force decay constant, α is the particle polarizability, *c* is the speed of light, and *I* is the local intensity of the laser light at the location the nanoparticle. Assuming the evanescent field behaves as *I = I*_max_ exp(−β*z*), we obtain the following expression for *z* after expanding about an average height above the waveguide, *z*_ave_:

[1]
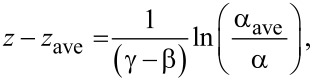


where α_ave_ is the average polarizability of a particle. The observed scattered light intensity from the nanoparticle, *P*_mic_*,* is given by *P*_mic_ *= B·I·*σ_scat_, where *B* is a constant dependent on the observation conditions (e.g., the numerical aperture of the lens, exposure time). Assuming Rayleigh theory, the scattering cross section σ_scat_ is proportional to α^2^, and by substitution we obtain:

[2]
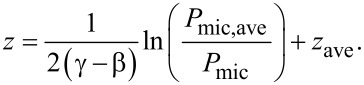


As the particle approaches the waveguide surface, *z* decreases and *P*_mic_ increases at a rate that is inversely proportional to γ, a measure of the coating strength. The more repulsive the interaction between the nanoparticle and waveguide, the higher the value of γ, representing a higher quality coating of the nanoparticle. Implicit in Equation 2 is that the variation in scattered light observed experimentally is due primarily to variations in σ_scat_, which in this case is due to differences in particle size as well as the particle’s dielectric constant, rather than the particle’s position in the evanescent field. If we expect variations in *z* to be ±10 nm, then with *I = I*_max_ exp(−β*z*), the variation in scattered light due to position is ≈10%, which is ≈100× less than observed in the experiment. In previous studies (Hristov et al., 2017), the particle material was held constant while varying the size, which permits analysis of particle size based on variations in *z* [24]. Since that approach is not possible with these particles due to the change in dielectric constant with particle coating, we instead used the observed scattered light to normalize propulsive forces and subsequently elucidated the effect of particle–waveguide interactions on velocity.

The propulsion force is the summation of scattering and absorption forces, *F*_prop_ = *F*_abs_ + *F*_scat_ = (σ_scat_ + σ_abs_)*I*/*c*, where σ_scat_ and σ_abs_ are the scattering and absorption cross section, respectively. The interaction between the particle and waveguide surface is represented by a friction force, *F*_fric_, which is a complex function of numerous dynamic properties. We use a simple approach, assuming *F*_fric_ is inversely proportional to height and increases linearly with velocity (*F*_fric_* = Av*/*z*, where *A* is a constant and *v* is the velocity). By equating *F*_fric_ and *F*_scat_:

[3]
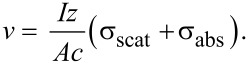


Combining *P*_mic_ *= B·I·*σ_scat_ with Equation 3 yields a relationship between the velocity and observed scattered light, which we expand about an average velocity *v*_ave_ as

[4]
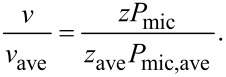


In Equation 4, we assumed that the ratio of σ_abs_/σ_scat_ varies less strongly than *y* and σ_scat_ (reflected in *P*_mic_). From Rayleigh theory, σ_scat_ varies with α^2^ while σ_abs_/σ_scat_ varies roughly with α. While this assumption will add noise, it should be small in comparison with the overall effect. For non-absorbing dielectric particles, the above result is exact. Since the propulsive force can be normalized to the observed light, this approach can be used to discriminate differences in particle–waveguide interactions independent of our knowledge of particle properties (e.g., size or refractive index) or how they explicitly vary in the sample.

**Figure 6:**
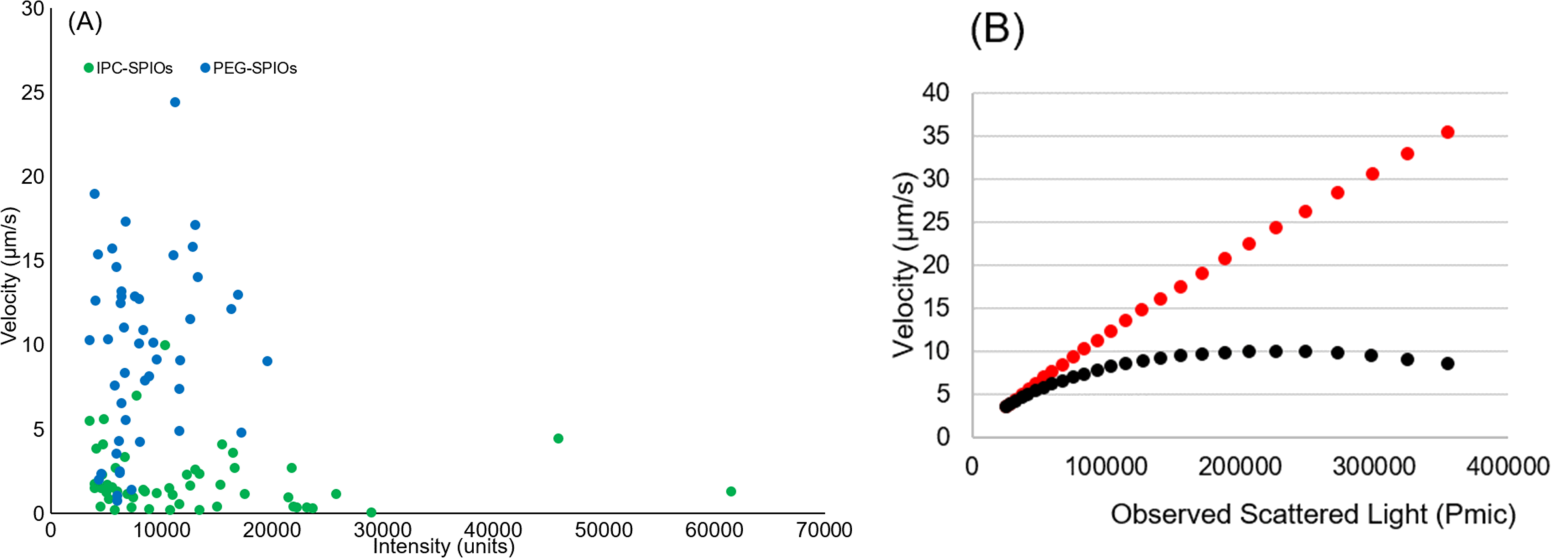
Velocity vs intensity. (A) Velocity of individual PEG- and IPC-coated SPIOs vs measured scattering intensity. (B) Predicted variation in the observed velocity for well coated (red) and poorly coated (black) particles from Equations 1–4 using γ = 0.2 nm^−1^, γ = 0.1 nm^−1^, and β = 0.01 nm^−1^ used for well coated, poorly coated, and evanescent field, respectively.

Figure 6A and Figure 6B show the measured and predicted variation in observed velocity of individual particles across the waveguide versus the intensity of scattered light, with the prediction resulting from Equations 1–4, using values of β = 0.01 nm^−1^ and γ *=* 0.1 nm^−1^ and 0.2 nm^−1^ [24]. PEG-SPIOs have a higher value of γ than IPC-SPIOs, meaning there is a greater repulsive force between the negatively charged waveguide and PEG-SPIOs. This repulsive force is related to particle surface chemistry and charge, particle size, and particle dielectric constant. The zeta potential and size data in Table 1 correspond to this conclusion, with the IPC-SPIOs having a zeta potential closer to neutral and a larger size than PEG-SPIOs. Given data obtained here, this system provided qualitative information on particle coating quality. However, this method needs to be refined further to accurately discern specific changes in particle size and surface properties.

### Analytical model for velocity vs solute concentration

Additionally, we have developed a preliminary model to describe the effect of the salt concentration (salinity) of the solution on a nanoparticle’s velocity across the waveguide. For spherical particles smaller than the wavelength of light [39–42], momentum transfer of incident photons triggers scattering and absorption forces exerted on the particles, directing particle movement along the laser waveguide [20]. To simplify the model, the particles are considered as non-absorbing dielectric particles, only optical scattering acts on the particle,

[5]
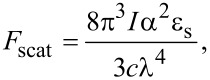


where the particle polarizability is α = [3*V*(ε − ε_s_)/(ε + 2ε_s_)], *V* is the particle volume, ε and ε_S_ are the dielectric constants of the particle and solution, respectively, and *I* is the local intensity.

As the particle travels in solution, a drag force also acts on the particle along the opposite direction of the laser waveguide. According to Stokes’s Law, the drag force can be written as,

[6]
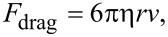


where η is the solution viscosity, *r* is the particle radius and *v* is the particle velocity.

When a particle enters the evanescent field and is trapped by the gradient force, it can be transported along the direction of the laser waveguide at the speed *v*. By equating the scattering force and the drag force, we obtain,

[7]
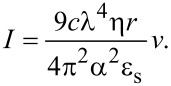


If we set *Q* = [(9*c*λ^4^η*r*) / (4π^2^α^2^ε_s_)], then Equation 7 can be rewritten as,

[8]
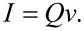


Based on the Jones–Dole equation, the solution viscosity η can be expressed by the solvent viscosity η_0_ and the solute concentration *C* as,

[9]
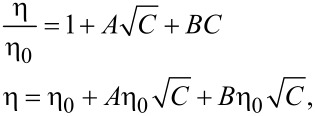


where *A* is a positive constant that depends on the electrostatic forces on the ions and *B* is the Jones–Dole coefficient, which is an empirical constant correlating ion–solvent interactions. In Equation 9, the solution viscosity η increases proportionally as the solute concentration *C* increases.

**Figure 7:**
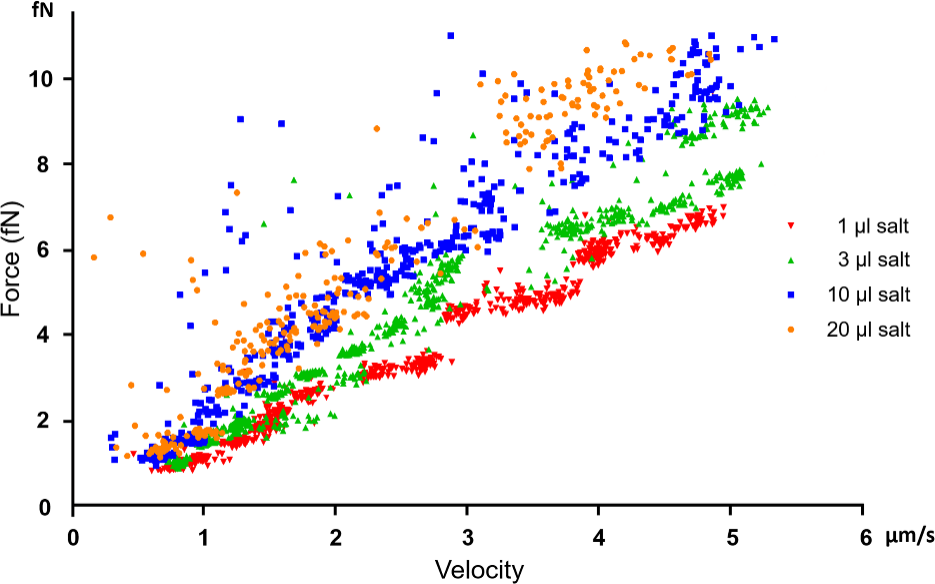
Measured velocity and drag force of the same particle in solution with different salt concentrations. For salt concentrations C1 > C2 > C3 > C4, the relation between particle velocity and measured drag force exhibits the relation as shown in the figure.

On the basis of the Stogryn model, the solution dielectric constant (permittivity), ε_s_, is inversely proportional to the solute concentration, *C* [43–44]*.* As the solute concentration *C* increases, the solution dielectric constant ε_s_ decreases, thus the particle polarizability α also decreases [45]. Taking the relation between the solute concentration *C* and the solution viscosity η, the solution dielectric constant ε_s_ and the particle polarizability α can be related in Equation 8, where *Q* is proportional to the solute concentration *C*. Additionally, using Equation 6, the drag force was calculated form the particle velocity and theoretical solution viscosity. The 10× PBS was approximated as a salt solution containing 1.37 M sodium chloride. Equation 6 was fitted using published empirical data [46]. The calculations assumed an average particle radius of 100 nm. For the same type of particle with given salt concentrations, 20 μL > 10 μL > 3 μL > 1 μL of 10× PBS, this trend agrees with our experimental data as shown in Figure 7.

## Conclusion

Near-field light scattering can be used to effectively measure changes in nanoparticle surface properties and size, as was demonstrated here using uncoated SPIOs, PEG-SPIOs, and IPC-SPIOs. The relationship between nanoparticle coating properties and the velocity of particles in either water or aqueous solutions with varying salt concentrations were explored. We described analytical models explaining the effect of surface–waveguide interactions on the velocity of particles across the waveguide in both water and salt-containing solutions. Although there is large scattering in the experimental data, the location of populations of particles in the velocity versus intensity plots generally agree with the analytical models; further refinement of the technique is necessary to develop a more reliable technique. Although not studied in the present work, this technique holds promise for dynamic studies of reaction kinetics at the particle surface that result in changes in particle size, dielectric constant, or surface chemistry, which is of particular interest in protein or biomolecule binding to nanoparticles for biomedical applications.

## Experimental

### Materials for nanoparticle synthesis

The metallic precursors iron(II) chloride (FeCl_2_), iron(III) chloride (FeCl_3_), *N*,*N*’-diisopropylcarbodiimide (DIC), 4-dimethylaminopyridine (DMAP), dimethyl sulfoxide (DMSO), dichloromethane (DCM), dimethylformamide (DMF), Pd black, sodium ascorbate, sodium carbonate (Na_2_CO_3_) and dextrans were purchased from Sigma-Aldrich (St. Louis, MO, USA). Ammonium hydroxide (28–30 wt %) was purchased from BDH (Poole, Dorset, UK). Poly(ethylene glycol) 6000 (PEG 6000) was purchased from Alfa Aesar (Heysham, UK). Sodium phosphate monobasic and dibasic were purchased from Amresco (Solon, OH, USA). Tribenzyloxybenzoic acid (TBBA) was purchased from Tokyo Chemical Industry. N_2_ purged deionized (DI) water was used throughout the entire experiment. All chemicals used were of analytical grade.

### Synthesis of pseudotannin polygallol

The synthesis of the pseudotannins consisted of conjugating benzyloxy benzoic acids (BBA) to a linear dextran backbone and converting those BBAs to phenols via a palladium (black) catalyzed deprotection reaction, creating a brush-like polyphenol [47–51]. The conjugation of BBAs used an optimized Steglich esterification to bind the carboxyl groups on the BBAs to the free hydroxyl groups on 40 kDa dextran. For this study, tribenzyloxybenzoic acid (TBBA) was used as the phenolic precursor, resulting in a polygallol polymer [35–37].

### Synthesis of interpolymer complexed (IPC)-SPIOs

One mmol FeCl_2_ was added dropwise to 2 mmol FeCl_3_ in 50 mL DI water under magnetic stirring [26,52–54]. The solution was heated to 80 °C, and 30 mL of 1 M NH_4_OH solution was added dropwise. After adding ammonia, the mixture was left for 5 h to approach completion. The solution was cooled to room temperature, sonicated for 1 h, and washed with DI water via centrifugation and magnetic decantation until the pH was around 8. The first 5 min of the centrifugation were done at 2,000 rcf followed by 10 min at 20,000, 35,000, and 50,000 rcf, respectively, for each of the three centrifugations. Finally, the nanoparticles were resuspended in DI water to create a 5 mg/mL solution.

For PEGylation, PEG 6 kDa was dissolved in DI water and mixed with suspended SPIOs (1 mg/mL) in a 1:1 ratio [33]. The particles were stirred for 24 h at 800 rpm and were separated via magnetic decantation; the supernatant was decanted. The solution was washed three times with DI water to remove excess polymer. PEG-SPIOs (1 mg/mL in water) and polygallol (1 mg/mL) were vortexed in a 1:1 volume ratio for 1 h. Finally, 1 M of pH 7.2 phosphate buffer solution was added into the mixture in a 9:1 ratio to convert the hydrogen bonds between PEG and polygallol for the complexation reaction.

### Transmission electron microscopy and energy dispersive X-ray spectroscopy

Transmission electron microscopy (TEM, JEOL JEM 2100F, Japan) was performed at a voltage of 200 kV to assess the size and morphology of the synthesized particles. Several drops of the diluted samples were placed on the surface of an ultrathin carbon film on lacey carbon support film grid (400 mesh, Ted Pella Inc., Redding, CA). During TEM imaging, energy dispersive X-ray spectroscopy (EDS) was used for a quantitative elemental analysis of uncoated SPIOs (Fe_3_O_4_). ImageJ software (NIH, Bethesda, MD) was used to measure the mean diameter of the particles from TEM images.

### Scanning electron microscopy

Uncoated SPIOs, PEG-SPIOs, and IPC-SPIOs were characterized by field-emission scanning electron microscopy (FE-SEM, Supra 55 VP, Carl Zeiss, Thornwood, NY) with an accelerating voltage of 2 kV. Each dried sample was placed on a metallic pin stub with adhesive and was coated with carbon (Cressington 208C High Vacuum Turbo Carbon Coater, Ted Pella Inc.).

### Dynamic light scattering and zeta potential

The hydrodynamic diameter, size distribution, polydispersity index (PDI), and zeta potential of each sample was determined by dynamic light scattering (DLS, Zetasizer, Malvern NanoZS, Worcestershire, UK) at room temperature. For DLS, all samples were suspended in water at a SPIO concentration of 0.05 mg/mL and placed in a DTS1070 folded capillary cuvette cell (Malvern Instruments, Worcestershire, UK).

### Near-field light scattering system (NLS)

A near-field evanescent field was generated by a single-mode Fabry–Pérot coherent laser diode that was coupled into a silicon nitride waveguide (Optofluidics) and was capable of trapping metallic nanoparticles in solution on the waveguide surface. A microscope with a high frame rate CMOS camera (1,500 fps, Basler acA2000-165 µm) was used to measure the intense scattered light (local intensity) from trapped metallic nanoparticles. A 1064 nm laser (NanoTweezer^TM^ instrument, Optofluidics, maximum 350 mW) was used to produce the evanescent field by coupling the laser beams into the waveguide with an optical fiber. The samples were drawn through the system and traveled through the nanophotonic trap. The flow direction of the sample is perpendicular to the length of the nanophotonic trap. The flow rate was restricted to 10 μL/min range using a proportional integral derivative (PID) controller and an in-line flow rate sensor. Nanoparticles that flow closer to the waveguide are usually trapped within the evanescent field and are pushed along the waveguide by laser radiation pressure. NLS custom software package was used to image and analyze the trapped nanoparticles by tracking each particle, measuring the particle density and generating the potential energy well analysis. In the salt concentration model, different concentrations of 10× PBS buffer in 10 mL DI water were used to investigate velocity and drag force (i.e., each of the groups are labelled with a volume that corresponds to the volume in μL of 10× PBS buffer in 10 mL water). In order to measure PEG-SPIOs, 1% pluronic (Pluronic F-68) surfactant was added to all solutions. Uncoated SPIOs were unstable under the same conditions as PEG-SPIOs and IPC-SPIOs, so we measured these particles in water.

### Waveguide and microfluidic chip

As previously described by Kong et al., the microfluidic chips are 1 cm^2^ silicon substrates with silicon nitride (Si_3_N_4_) waveguides patterned by standard microfabrication techniques [38]. The waveguide is composed of rectangular cross-sections with height of 250 nm and width 600 nm, which are cladded by SiO_2_ (3 μm above and 5 μm below) across the whole chip except for a 200 × 200 μm experimental window in the center region of the chip (Figure 1). During the experiment, the topmost cladding layer is removed, permitting interaction between the samples and exposed waveguide. Each chip is comprised of three waveguides, which allows for experimental redundancy. The distance between each waveguide at the input edge is 1 mm and after the experiment window, the waveguide converges to a corner of the chip near the output edge where transmitted light is collected by a photodetector. Three polarization-maintaining optical fibers are supported by a silicon v-groove array and are optically aligned to the three waveguides permanently bonded to the chips. Each chip has two through-holes for fluidic access. Fluid lines are attached to the chip through the o-rings that are sealed around the through-holes on the back of the chip. Therefore, flow control is attained through a pressure-regulated vacuum pump. The microfluidic chips can be reused by washing with buffer of 1% pluronic F-68 surfactant in 0.22 μm filtered deionized water.
